# The MLK-1/SCD-4 Mixed Lineage Kinase/MAP3K functions to promote dauer formation upstream of DAF-2/InsR

**DOI:** 10.17912/micropub.biology.000405

**Published:** 2021-06-15

**Authors:** Neal R Rasmussen, Harold E Smith, David J Reiner

**Affiliations:** 1 Texas A&M Health Science Center; 2 National Institute of Diabetes and Digestive and Kidney Diseases

## Abstract

The *C. elegans *dauer is an alternative third stage larva induced by dense population and adverse environmental conditions. Genes whose mutants caused dauer formation constitutive (Daf-c) and dauer formation defective (Daf-d) phenotypes were ordered via epistasis into a signaling network, with upstream DAF-7/TGF-beta and DAF-11/receptor guanylyl cyclase defining sensory branches and downstream DAF-2/Insulin receptor and DAF-12/nuclear hormone receptor executing the dauer decision. Mutations in the Scd genes were defined as incompletely penetrant suppressors of the constitutive dauer phenotype conferred by mutation of the DAF-7/TGF-beta signaling axis. SCD-2 was previously shown to be an ortholog of mammalian ALK (Anaplastic Lymphoma Kinase), a receptor tyrosine kinase. Mutations disrupting the HEN-1/Jeb ligand, SOC-1/DOS/GAB adaptor protein and SMA-5/ERK5 atypical MAP Kinase caused Scd phenotypes similar to that of mutant SCD-2. This group regulated expression from a TGF-beta-responsive GFP reporter. Here we find that a strain harboring a mutation in the uncharacterized SCD-4 is mutant for MLK-1, the *C. elegans *ortholog of mammalian Mixed Lineage Kinase and *Drosophila slipper *(*slpr*), a MAP3 kinase. We validated this finding by showing that a previously characterized deletion in MLK-1 caused a Scd phenotype similar to that of mutant SCD-4 and altered expression from the TGF-beta-responsive GFP reporter, suggesting that SCD-4 and MLK-1 are the same protein. Based on shared phenotypes and molecular identities, we hypothesize that MLK-1 functions as a MAP3K in the SCD-2/ALK cascade that signals through SMA-5/ERK5 MAP Kinase to modulate the output of the TGF-beta cascade controlling dauer formation in response to environmental cues.

**Figure 1. Mutant MLK-1/SCD-4 MAP3K suppresses dauer constitutive mutations in a manner similar to the mutant SCD-2/ALK Receptor Tyrosine Kinase:**
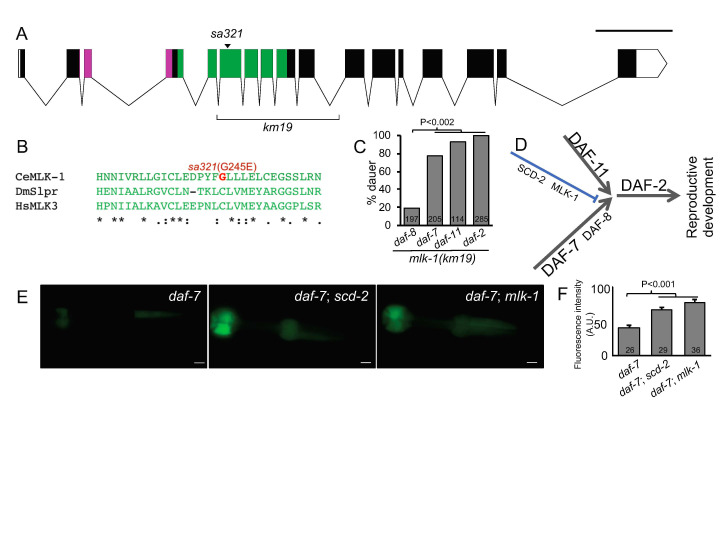
**A)** The gene model of *mlk-1/scd-4*. Pink = SH3 domain, green = S/T kinase domain. Scale bar = 1000 bp. The *scd-4(sa321)* mutation causes a G245E change in the kinase domain (also see B, below). The *km19* deletion of the kinase domain (Mizuno, 2004) is indicated by a bracket. **B)** A subset of an alignment of kinase domains of *C. elegans* (Ce) MLK-1 harboring the G245E mutation (red), *Drosophila* (Dm) *slpr* and human (Hs) MLK3. **C)** The *mlk-1(km19)* deletion confers a genetic “interaction fingerprint” similar to that of mutations in *scd-2*, *hen-1, soc-1* and *sma-5* and replicates phenotypes conferred by *scd-4(sa321)* (Reiner *et al.*, 2008). Shown is percent dauer formation at 25˚C with the set of reference mutations *daf-8(e1393)*, *daf-7(e1372)*, *daf-11(m47)* and *daf-2(e1370)* used previously: strong suppression of the Daf-c phenotype conferred by *e1393*, moderate suppression of *e1372*, weak suppression of *m47* and no suppression of *e1370*. Not shown are the Daf-c single mutants assayed in parallel, which were all 100% dauer at 25˚C. P< 0.0001, <0.0001, =0.002 and n.s., respectively (Chi square with Yates correction). *mlk-1(km19)* and N2 wild type yielded no dauers when scored in parallel at 25˚C. **D)** A schematic of major signaling axes in the dauer regulatory network that are mutated to a dauer constitutive (Daf-c) phenotype. DAF-11/receptor guanylyl cyclase and DAF-7/TGF-beta define major sensory inputs. DAF-8/Smad is lesser player downstream of DAF-7/TGF-beta, and is mutated to a weaker Daf-c phenotype. By genetic interactions, DAF-2/InsR functions downstream of the DAF-7 and DAF-11 axes. **E)** Pharyngeal GFP expression from the DAF-3/co-Smad-responsive reporter *cuIs2* was previously shown to be suppressed by mutation of DAF-7/TGF-beta (Thatcher *et al.*., 1999; Reiner *et al.*, 2008; left panel) and was restored by additional mutation of SCD-2/ALK (center) and MLK-1/SCD-4/MAP3K (right panel). Scale bar = 10 µm. **F)** Quantified pixel intensity of pharyngeal GFP signal from *cuIs2* in the *daf-*7 single mutant and double mutants with *scd-2* and *mlk-1*, respectively,measured as arbitrary units on a Nikon eclipse Ni epifluorescent microscope with DS-Fi2 camera (Nikon) and NIS Elements Advanced research, version 4.40 (Nikon). Error bars represent std error and P value was calculated by ANOVA.

## Description

The *C. elegans* dauer is an alternative L3 stage larva that forms under harsh environmental conditions, including low food, high temperature, and high concentration of constitutively secreted dauer pheromone. Genetic screens identified genes conferring dauer-constitutive and dauer-defective phenotypes (Daf-c and Daf-d, respectively; Hu, 2007). Double mutant analysis using principles of epistasis and parallelism ordered genes controlling the dauer process into a network (Gottlieb and Ruvkun, 1994; Thomas *et al.*, 1993). Molecular genetic cloning of genes provided identities with similarity to orthologs in *Drosophila* and mammals. Taken together, these approaches arrived at a model of four main signaling axes controlling entry into dauer: upstream and parallel TGF-beta (DAF-7, mutated to Daf-c) and receptor guanylyl cyclase (DAF-11, mutated to Daf-c) signals reflect parallel processing by sensory neurons, revealed by laser ablation experiments (Birnby *et al.*, 2000; Ren *et al.*, 1996; Schackwitz *et al.*, 1996). Downstream, serial Insulin/IGF-like growth factor receptor (DAF-2, mutated to Daf-c; (Kimura *et al.*, 1997) and nuclear hormone receptor (DAF-12/NHR, mutated to Daf-d; (Antebi *et al.*, 2000) signals control and execute tissue-specific changes in the animal (**Fig. 1C**, DAF-12 not shown). Mutants for each signaling axis also control diverse developmental and metabolic outputs in addition to the dauer decision. The four-axis model of signaling control of dauer formation neglects potential positive- and negative-feedback loops and is thus likely reductive. Still, these approaches have provided a robust framework for further investigation into the control of the dauer developmental decision by sensory and endocrine signaling modalities.

Genetic screens for mutations that suppress the Daf-c phenotypes conferred by mutations in the DAF-7/TGF-beta signaling cascade identified expected proteins that confer Daf-d phenotypes when mutated (Inoue and Thomas, 2000). Mutants for transcription factors downstream in the TGF-beta signal, DAF-3/co-Smad and DAF-5/Sno-ski (da Graca *et al.*, 2004; Patterson *et al.*, 1997; Tewari *et al.*, 2004), completely suppress Daf-c mutations in the TGF-beta signal. The DAF-16/FoxO transcription factor, downstream of DAF-2/InsR, confers a partial dauer phenotype in double mutant combinations with Daf-c components of TGF-beta signaling (Lin *et al.*, 1997; Ogg *et al.*, 1997). And mutations in DAF-12/NHR, thought to be the most downstream player in the dauer regulatory network, completely suppress mutations in the TGF-beta group that confer a Daf-c phenotype.

Yet this screen also identified mutations – suppressors of constitutive dauer (Scd) – that partially but not completely suppressed the Daf-c phenotype of mutant TGF-beta group genes. These mutations defined three novel genes: *scd-1*, *scd-2* and *scd-3* (Inoue and Thomas, 2000). One of these, *scd-2*, encodes a protein orthologous to Anaplastic Lymphoma Kinase (ALK), a receptor tyrosine kinase that in humans is a proto-oncogene (Reiner *et al.*, 2008). SCD-2/ALK and its putative growth factor ligand HEN-1/Jeb also regulate diverse sensory signals (Ishihara *et al.*, 2002; Kitazono *et al.*, 2017; Shinkai *et al.*, 2011; Wolfe *et al.*, 2019). Mutations in *scd-2* at 25˚C strongly but not completely suppressed the DAF-c phenotype of mutant DAF-8/R-Smad, moderately suppressed the Daf-c phenotype of mutant DAF-7/TGF-beta, weakly but consistently suppressed the Daf-c phenotype of mutant DAF-11/rGC, and failed to suppress the Daf-c phenotype of mutant DAF-2/InsR. Through screening to test whether mutations in candidate genes conferred Scd interactions similar to those of mutant SCD-2/ALK, its putative ligand HEN-1/Jeb, adaptor protein SOC-1/DOS/GAB, and ERK5/MAP Kinase SMA-5 were also identified as conferring similar Scd phenotypes. This model was further supported by showing that expression from a transgenic promoter::GFP fusion repressed by DAF-3/Co-Smad (Thatcher *et al.*, 1999) was regulated by mutations in the putative SCD-2/ALK signaling cascade. Taken together, these results suggested that SCD-2/ALK and functionally related genes that confer a similar Scd phenotype when mutated collaborate with the main DAF-7/TGF-beta cascade to co-regulate dauer-regulating genes throughout the animal (Reiner *et al.*, 2008).

A fourth Scd gene, *scd-4*, was defined by a single allele, *sa321*, which mapped to chromosome V and thus may have been allelic with *scd-2 or soc-1*. We previously mapped the phenotype of suppression of *daf-7* phenotype of *sa321* to the *unc-62*–*dpy-11* interval on chromosome V, excluding the possibility that *sa321* was an allele of *scd-2*, which is located to the right of *dpy-11*. *sa321* also complemented the suppression phenotypes of *soc-1(n1789)* and *scd-2(y386)*, suggesting that *sa321* defined a novel Scd gene*, scd-4* (Reiner *et al.*, 2008). *sa321* does not alter other phenotypes on plates conferred by mutations in TGF-beta like *daf-7(e1372)*: egg-laying defective (Egl), clumping on the plate (Cpy), and dark intestine (Din; not shown; Thomas *et al.*, 1993).

We sequenced the whole genome of the strain JT7478 *daf-8(sa234)*; *scd-4(sa321)*. In the genetic interval of *unc-62*–*dpy-11*, we identified non-synonymous mutations in three genes: a G245E mutation in *mlk-1*, an A189G mutation in *ncx-2*, and an I5498V mutation in *ttn-1*. Of these, MLK-1, a MAP3 Kinase most similar to human MLK3 (mixed lineage kinase) and *Drosophila slipper* (*slpr*) functions in signal transduction and was a parsimonious candidate for a Scd gene based on our knowledge of SCD-2 as a receptor tyrosine kinase. With Sanger sequencing we confirmed the DNA lesion in *mlk-1* of G734A, resulting in the G245E amino acid change in the Ser/Thr kinase domain of MLK-1 (**Fig. 1A, B**).

To validate that *scd-4* is actually *mlk-1*, we used a deletion allele in *mlk-1*, *km19* (Mizuno *et al.*, 2004). As with mutations in *hen-1* and *scd-2*, *sa321* and *km19* mutant animals are superficially wild-type when grown on plates. Like *sa321* and mutations in *scd-2*, at 25˚C *km19* causes strong but not complete suppression of mutant *daf-8*, moderate suppression ofmutant *daf-7*, very weak suppression of mutant *daf-11*, and no suppression of mutant *daf-2* (seeabove; **Fig. 1C**). Also like *scd-2(y386)*, *mlk-1(km19)* restored GFP expression from the *cuIs2* reporter repressed by *daf-7(e1372)* (**Fig. 1E**, quantified in **1F**). Furthermore, as observed with certain mutated components of the putative SCD-2/ALK cascade, *sa321* and *km19* do not alter the Egl, Cpy and Din phenotypes of *daf-7(e1372)*, suggesting that the interaction between MLK-1 and DAF-7/TGF-beta signaling is specific to dauer formation. Thus, we conclude that *scd-4(sa321)* is an allele of *mlk-1*. Given their shared mutant phenotypes and identity as signaling molecules, we hypothesize that MLK-1/MAP3K functions downstream of SCD-2/ALK to regulate dauer formation in conjunction with TGF-beta (**Fig. 1D**), but not other TGF-beta-dependent phenotypes.

## Methods

**Animal assays**

Animals were grown under standard growth conditions at 15˚C or 25˚ for growth or dauer assays, respectively. Percentage dauer formation was determined from synchronized broods grown at 25˚C laid by parents grown at 15˚C. All assays to be compared were grown in parallel. Response of pharyngeal GFP levels expressed from the *cuIs5[P_myo-2-C-subelement_::gfp]* to mutational state was as described (Reiner *et al.*, 2008) except for image capture and quantification (see below). These animals were all grown at 15˚C.

**Microscopy**

Reporter fluorescence was recorded on a Nikon eclipse Ni epifluorescence microscope with DS-Fi2 camera (Nikon) and NIS Elements Advanced research, version 4.40 (Nikon). Images were captured at the same settings and a uniform exposure time of 60 msec with the 40x objective.

**Whole genome sequencing**

The strain JT7478 *daf-8(sa234)*; *scd-4(sa321)* was subjected to whole-genome sequencing (50-bp single-end reads, 20-fold genome coverage). Candidate mutations (homozygous, nonsynonymous variants) were identified using a previously described pipeline (Smith and Yun, 2017) and annotated using ANNOVAR (Yang and Wang, 2015).

## Reagents

**Animal strains used**

CB1383 *daf-8(e1383)* I

DV3650 *daf-8(e1383)* I; *mlk-1(km19)* V

CB1372 *daf-7(e1272)* I

DV3664 *daf-7(e1272)* I; *mlk-1(km19)* V

MT4304 *daf-11(m47)* V

DV3779 *mlk-1(km19) daf-11(m47)* V

TY1614 *unc-62(e644)* *dpy-11(e224)* V

DV3659 *unc-62(e644)* *dpy-11(e224) daf-11(m47)* V

CB1370 *daf-2(e1370)* III

DV3773 *daf-2(e1370)* III; *mlk-1(km19)* V

OK43 *cuIs5[P_myo-2-C-subelement_::gfp]* I

TY3862 *cuIs5[P_myo-2-C-subelement_::gfp]* I; *daf-7(e1372)* III

TY3883 *cuIs5[P_myo-2-C-subelement_::gfp]* I; *daf-7(e1372)* III; *scd-2(y386)* V

DV3855 *cuIs5[P_myo-2-C-subelement_::gfp]* I; *daf-7(e1372)* III; *mlk-1(km19)* V
